# Multidimensional Functional Phenotyping in Children with Joubert Syndrome: A Pilot Case Series

**DOI:** 10.3390/brainsci16030305

**Published:** 2026-03-12

**Authors:** Łukasz Mański, Aleksandra Moluszys, Anna Góra, Eliza Wasilewska, Agnieszka Rosa, Krzysztof Szczałuba, Krystyna Szymańska, Jolanta Wierzba

**Affiliations:** 1 Gdansk Medical Academy of Applied Sciences, 80-335 Gdansk, Poland; aleksandra.moluszys@wsz.pl (A.M.);; 2 Department of Allergology, Medical University of Gdansk, 80-210 Gdansk, Poland; 3 Center of Excellence for Rare and Undiagnosed Disorders, Medical University of Warsaw, 02-091 Warsaw, Polandkrzysztof.szczaluba@wum.edu.pl (K.S.); 4 Department of Pediatric Neurology and Rare Disorders, Medical University of Warsaw, 02-091 Warsaw, Poland; 5 Department of Internal and Pediatric Nursing, Institute of Nursing and Midwifery, Medical University of Gdansk, 80-208 Gdansk, Poland

**Keywords:** Joubert syndrome, motor development, postural control

## Abstract

**Highlights:**

**What are the main findings?**

• Children with Joubert syndrome in this exploratory cohort demonstrated postural control scores that did not parallel gross motor performance levels, suggesting that axial control may constitute a distinct descriptive functional dimension within this sample.
• Variability in thoracoabdominal configuration and relatively limited thoracic excursion were descriptively observed alongside postural control limitations and may be compatible with altered respiratory–postural integration.

**What are the implications of the main findings?**

• Global gross motor outcome measures alone may not fully capture multidimensional functional characteristics potentially linked to cerebellar network involvement.
• A multidimensional assessment framework incorporating axial and thoracoabdominal domains may support hypothesis generation regarding network-level functional organization in Joubert syndrome.

**Abstract:**

**Background/Objectives:** Joubert syndrome is a rare neurodevelopmental disorder characterized by congenital cerebellar and brainstem malformations affecting networks involved in predictive motor control, sensorimotor integration, and autonomic regulation, resulting in a heterogeneous motor phenotype. Functional impairment is typically described using global gross motor scores, which may not adequately reflect axial control, postural organization, musculoskeletal alignment, or respiratory–postural interactions. The objective of this descriptive pilot case series was to provide a multidimensional functional characterization of children with Joubert syndrome by integrating standardized motor assessments with postural, musculoskeletal, and thoracoabdominal measures. **Methods:** Six children with genetically and radiologically confirmed Joubert syndrome underwent a single standardized assessment session conducted by the same examiner. This cross-sectional, non-controlled study was based on feasibility sampling, and no a priori power calculation was performed. Gross motor function and postural control were evaluated using the Gross Motor Function Measure-88 and the Balance Assessment Rating Scale. Additional measures included joint range of motion, sacral inclination angle, thoracic configuration, thoracic excursion during quiet breathing, and respiratory rate. Analyses were limited to descriptive statistics. **Results:** Gross motor performance varied widely across participants, whereas postural control scores did not parallel gross motor performance levels within the cohort. Inter-individual variability was observed in joint mobility, pelvic alignment, and thoracoabdominal configuration, including among children with relatively preserved gross motor scores. Thoracic excursion during quiet breathing demonstrated a relatively narrow and low within-cohort range. **Conclusions:** In this small exploratory case series, functional characteristics observed in this cohort extended beyond global motor scores. Axial control, postural organization, and thoracoabdominal configuration may represent relevant descriptive domains of functional presentation within a multidimensional framework. Larger, longitudinal, and controlled studies are required to determine their clinical and neurodevelopmental significance.

## 1. Introduction

Joubert syndrome (JS) is a rare neurodevelopmental ciliopathy characterized by congenital malformations of the cerebellar vermis and brainstem. The hallmark radiological feature is the molar tooth sign observed on magnetic resonance imaging (MRI) [1,2]. These structural abnormalities are known to affect cerebellar-brainstem circuits implicated involved in predictive motor control, axial stability, respiratory rhythm regulation, and sensorimotor integration [3,4]. As a result, children with JS present with a heterogeneous and developmentally complex motor phenotype.

**Figure 1:**
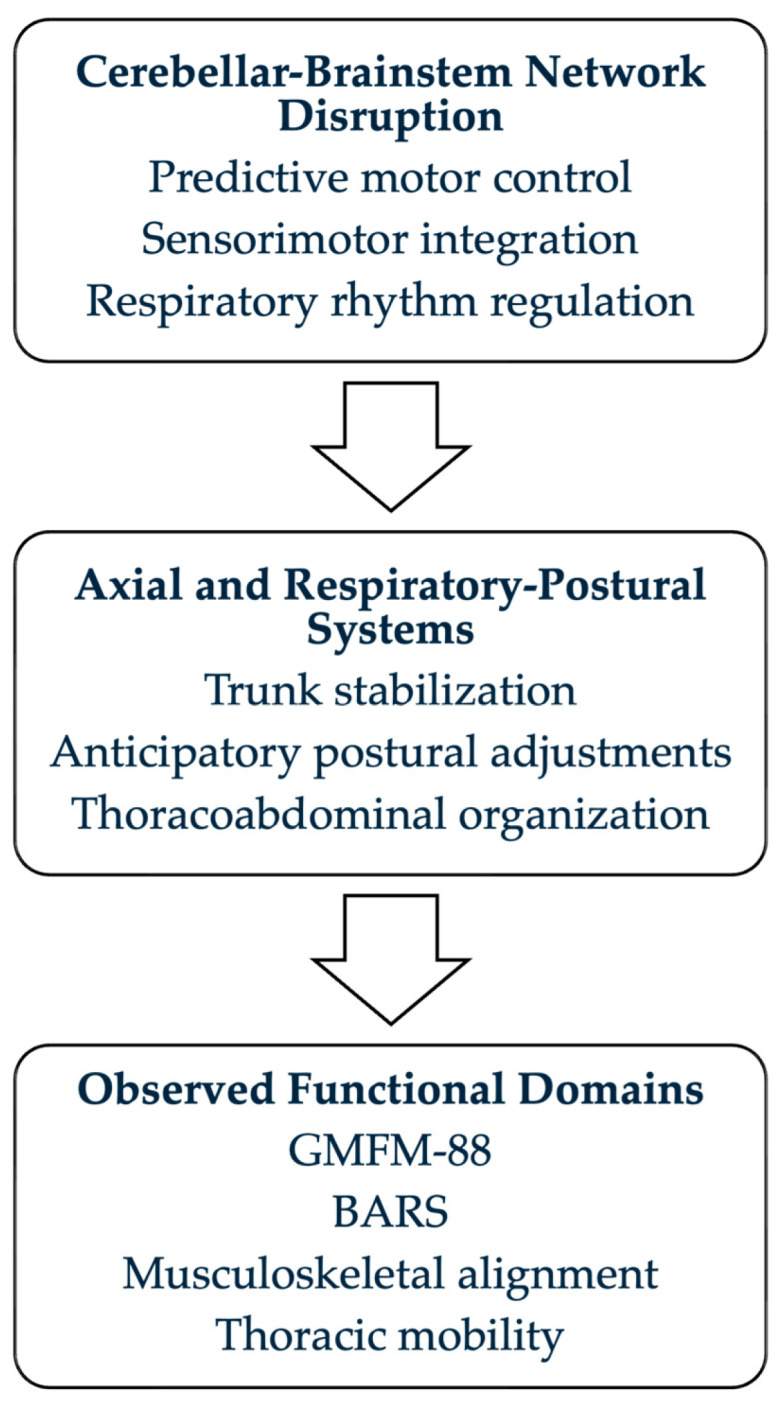
Conceptual framework of multidimensional functional organization in Joubert syndrome. Cerebellar-brainstem network disruption is hypothesized to influence axial and respiratory–postural systems, reflected in the observed functional domains assessed in this study.

Motor impairment in JS is most commonly described in terms of delayed developmental milestones and reduced gross motor scores [1,5]. However, cerebellar dysfunction extends beyond distal coordination and likely influences axial control and anticipatory postural adjustments [4,6]. From a developmental motor control perspective, cerebellar circuits contribute to predictive timing and internal model formation that support feedforward regulation of posture preceding voluntary movement [7]. Early disruption of cerebellar-brainstem connectivity may therefore influence trunk activation patterns, intersegmental coordination, and the integration of respiratory rhythm with postural stabilization. This framework provides a neurophysiological basis for considering axial and thoracoabdominal organization as core components of motor development in JS [8,9] (Figure 1).

Clinically, children with JS frequently demonstrate impaired trunk stabilization and altered proximal-distal motor coupling [10,11]. These features often coexist with atypical thoracoabdominal configuration and altered respiratory mechanics, suggesting disturbed integration between brainstem respiratory centers and axial postural control systems [12,13,14].

Despite these neurodevelopmental considerations, rehabilitation-oriented research in JS remains limited. Existing studies primarily consist of single case reports or small heterogeneous cohorts, and functional outcomes are typically assessed using generic tools such as the Gross Motor Function Measure-88 (GMFM-88) or the Pediatric Evaluation of Disability Inventory (PEDI) [15,16]. While these instruments quantify global performance, they do not capture potentially relevant dimensions linked to cerebellar network dysfunction, including axial organization or respiratory–postural integration. To date, no integrated framework has systematically examined how global motor performance, musculoskeletal alignment, and thoracic configuration coexist within individual functional profiles in children with JS [15].

The primary objective of this pilot case series was to provide a structured multidimensional characterization of axial and thoracoabdominal functional organization in children with Joubert syndrome. A secondary objective was to explore descriptive associations between axial motor function and thoracic configuration within an integrated assessment framework. We aimed to examine whether multidimensional assessment might reveal functional variability not fully reflected by global gross motor scores. The study was designed as an exploratory descriptive case series and was not intended to test causal mechanisms or evaluate intervention effects.

## 2. Materials and Methods

### 2.1. Study Design and Setting

This study was conducted as a descriptive, cross-sectional, non-controlled case series. The investigation was prospective in nature and involved a single standardized assessment session per participant. No randomization or blinding procedures were implemented.

Assessments were performed between June 2025 and September 2025 in two settings: during a structured assessment session at a national family meeting for children with Joubert syndrome and their caregivers, and at a pediatric neurorehabilitation outpatient clinic. Each participant completed a single standardized evaluation conducted by the same experienced pediatric physiotherapist.

The sample size was determined by feasibility and access to children with genetically confirmed Joubert syndrome during the study period. Given the exploratory and descriptive aim of this pilot study, no a priori hypotheses were formulated and no statistical power calculation was performed, as the study was not designed to test inferential hypotheses.

### 2.2. Participants

Six children with a confirmed diagnosis of Joubert syndrome were included in this descriptive case series. Diagnosis was established based on magnetic resonance imaging demonstrating the molar tooth sign and was supported by molecular genetic testing when available.

Inclusion criteria were: (1) confirmed diagnosis of Joubert syndrome; (2) age between 2 and 16 years; and (3) clinical stability allowing completion of standardized assessment. Exclusion criteria comprised: (1) severe cardiorespiratory instability; (2) acute orthopedic injury; (3) orthopedic surgery within the preceding six months; and (4) inability to complete any component of the assessment protocol. These criteria were applied to ensure reliable assessment and to minimize potential confounding effects on motor and respiratory measures.

Participants were recruited consecutively through collaboration with a National Association of Families of Children with Joubert Syndrome. The association organizes annual family meetings attended by families from across Poland. During one such meeting, eligible children were invited to participate in a structured assessment session conducted on-site. Additionally, families residing locally were informed about the study through the association and were invited to attend a standardized assessment session at a pediatric neurorehabilitation outpatient clinic. All assessments were conducted under identical conditions and according to the same protocol. Recruitment was based on availability during the study period, and all children meeting the predefined inclusion criteria were invited to participate. Participation in a family-meeting setting may have introduced selection bias toward families with higher levels of engagement in organized support activities.

Demographic and anthropometric data, including age, sex, height, and body weight, were recorded at the time of assessment.

### 2.3. Ethical Considerations

The study was conducted in accordance with the principles of the Declaration of Helsinki. Ethical approval was obtained from the Bioethics Committee of the Medical University of Gdansk (Resolution No. KB/194/2025; date of approval: 9 May 2025). Written informed consent was obtained from the parents or legal guardians of all participants prior to data collection. All data were anonymized before analysis and publication.

### 2.4. Functional Motor Assessment

Gross motor function was evaluated using the Gross Motor Function Measure-88 (GMFM-88). Postural control was assessed using the Balance Assessment Rating Scale (BARS). BARS was selected based on construct alignment with axial coordination and postural instability, domains consistently described as central features of the motor phenotype in Joubert syndrome. Although not disease-specific to JS, the scale captures functional manifestations of impaired balance, trunk control, and coordination across a broad spectrum of abilities. Segmental trunk control measures were considered; however, many such tools require a minimum level of independent sitting or are primarily focused on static segmental control, which may limit applicability across heterogeneous functional levels. In contrast, BARS allows standardized evaluation of both static and dynamic postural tasks and was therefore deemed more suitable for capturing global axial control characteristics within this small and functionally diverse cohort. Assessments were conducted by a single experienced pediatric physiotherapist following standardized procedures. The examiner was not blinded to diagnosis. Evaluations were performed in a quiet clinical environment, and rest periods were provided as needed to minimize fatigue. Total GMFM-88 and BARS scores were recorded for each participant. No functional assessment tools have been specifically validated for Joubert syndrome. Therefore, the assessment battery was selected based on construct relevance to theoretically grounded domains, including axial control, musculoskeletal organization, and respiratory–postural integration, rather than on syndrome-specific validation.

### 2.5. Postural and Musculoskeletal Assessment

Postural and musculoskeletal assessment included evaluation of hip, shoulder, and ankle range of motion, as well as measurement of the sacral inclination angle. Joint range of motion was measured using a handheld goniometer using standardized anatomical landmarks. Assessments were performed in standardized prone, supine, or standing positions, depending on the joint and parameter evaluated. External rotation of the shoulder and hip joints was assessed bilaterally due to their role in axial stabilization and trunk control. For hip extension, a single value was reported for each participant, representing the more restricted side, as the most limited range is considered to have the greatest influence on pelvic alignment and trunk posture. All angular measurements were performed twice within the same session by the same examiner, and the average value was used for descriptive analysis.

### 2.6. Thoracoabdominal Measures

Thoracoabdominal mobility was assessed using chest and abdominal circumferences, thoracic expansion during quiet breathing, and selected thoracic angular parameters, including subcostal, sternoclavicular, and posterior rib angles. All thoracoabdominal measures were included as descriptive indicators of relative thoracic configuration rather than normative or diagnostic parameters. Circumferential measurements were obtained at standardized anatomical landmarks using a flexible measuring tape and recorded at end-inspiration and end-expiration during spontaneous breathing, with thoracic excursion calculated as the difference between phases. Respiratory rate during quiet breathing was recorded as a descriptive contextual variable and counted manually over a 60 s period following a rest phase, with participants positioned comfortably. Thoracic angular measurements were obtained using a handheld goniometer in standardized prone positions and recorded descriptively without side-to-side comparative analysis. Thoracoabdominal angular and circumferential measurements were also performed twice within the same session, and the average value was used for analysis. To our knowledge, standardized pediatric normative reference values for surface-based thoracic angular measurements are not currently available in the literature. Although surface angular measurements have been described in postural and biomechanical research, their pediatric standardization remains limited, particularly in rare neurodevelopmental populations. Therefore, these parameters were interpreted as descriptive geometric indicators within the cohort rather than as normative deviations.

The selected measures collectively map onto multiple domains of the International Classification of Functioning (ICF), including body functions (postural and respiratory control), body structures (musculoskeletal alignment), and activity-level performance (gross motor function), supporting the multidimensional coherence of the assessment framework.

### 2.7. Statistical Analysis

Statistical analyses were performed using Jamovi (version 2.7.12; The Jamovi Project, Sydney, Australia). Given the exploratory design and small sample size, analyses were limited to descriptive statistics. Continuous variables are presented as means, standard deviations, medians, and ranges, while categorical variables are reported as frequencies and percentages. No inferential or hypothesis-driven analyses were conducted. Intra-rater measurement consistency was evaluated using repeated measurements obtained within the same assessment session. Absolute mean differences and ranges between first and second measurements were calculated for all musculoskeletal and thoracoabdominal parameters. Intraclass correlation coefficients (ICC) were explored; however, due to the very small sample size and limited variance in several parameters, ICC estimates were statistically unstable and were therefore not interpreted. All assessments were conducted by a single experienced examiner. While this approach reduces inter-rater variability, it may introduce potential systematic measurement bias, particularly for exploratory thoracoabdominal angular parameters that are not widely standardized in pediatric biomechanical assessment. These measures should therefore be interpreted as descriptive within-cohort indicators rather than externally validated biomechanical metrics.

## 3. Results

### 3.1. Participant Characteristics

The study cohort comprised six children with Joubert syndrome. The mean age of participants was 7.2 ± 3.3 years (range: 2–11 years). Demographic, anthropometric, and clinical characteristics of the participants are summarized in Table 1. All children demonstrated the characteristic molar tooth sign on magnetic resonance imaging. In addition, molecular genetic testing, when available, supported the diagnosis of Joubert syndrome. The cohort covered a broad pediatric age range and reflected the clinical heterogeneity typical of the disorder. All participants completed the full standardized assessment protocol, and no adverse events were observed during data collection.

### 3.2. Gross Motor Function and Postural Control

Gross motor performance, assessed using the GMFM-88 total score, demonstrated substantial variability across participants (mean 59.3 ± 32.4%, median 77.3%, range 10.2–84.5%). Postural control scores, evaluated using the BARS, did not parallel gross motor performance levels within the cohort and ranged from 4 to 28 (mean 12.2 ± 9.4; median 8.5). This pattern suggests that postural control limitations were present even in children with relatively preserved gross motor function. No consistent relationship between GMFM-88 and BARS scores was apparent at the descriptive level. Gross motor function and postural control outcomes are summarized in Table 2.

### 3.3. Postural and Musculoskeletal Measurements

Postural and musculoskeletal measurements demonstrated variability across participants. Differences were observed in joint range of motion parameters and sacral inclination angle within the cohort. Ankle dorsiflexion values ranged from 0° to 28°, while hip extension values ranged from 0° to 20° across participants. Shoulder range of motion values varied between children. Sacral inclination angle ranged from 15° to 25° within the cohort. Postural and musculoskeletal variability was observed across participants representing different levels of gross motor function. Detailed measurements are presented in Table 3.

### 3.4. Thoracoabdominal Measurements

Thoracoabdominal measurements varied across participants. Thoracic excursion during quiet breathing ranged from 0.5 to 2.0 cm and demonstrated a relatively low and narrow within-cohort range. Subcostal angle values ranged from 85° to 145°. Sternoclavicular and posterior rib angles also differed between participants. Respiratory rate during quiet breathing ranged from 12 to 18 breaths per minute. Detailed thoracoabdominal measurements are presented in Table 4. These parameters should be interpreted as relative within-cohort descriptors of thoracoabdominal configuration rather than clinically validated diagnostic metrics.

**Figure 2:**
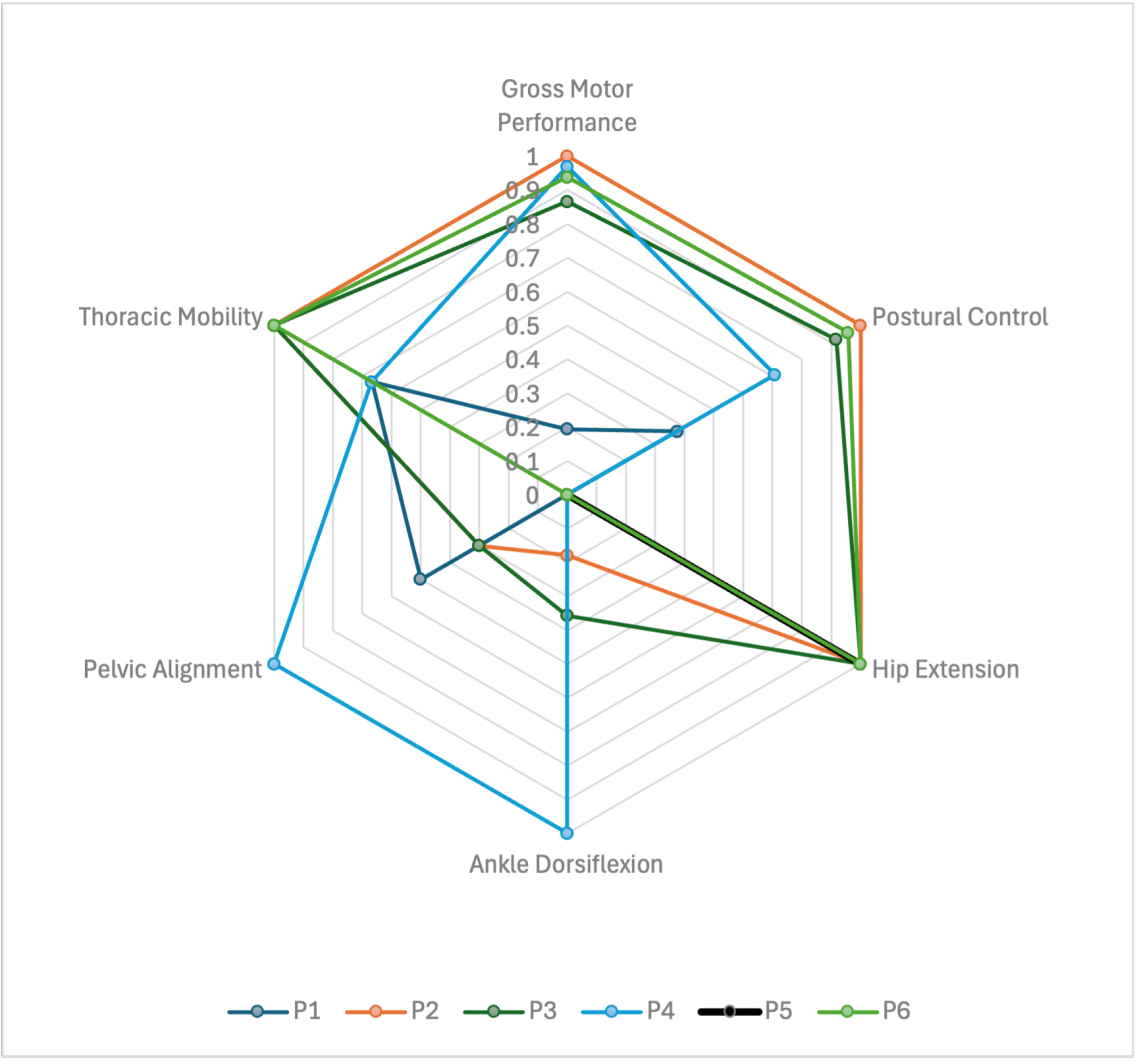
Multidimensional functional profiles of children with Joubert syndrome. Individual participant profiles (P1–P6) across selected functional domains. Variables were normalized within the cohort using min–max scaling (0–1 range) for visualization purposes only.

When individual profiles were examined descriptively, contrasting multidimensional patterns became apparent within the cohort (Figure 2). For example, participants with relatively preserved gross motor performance demonstrated comparatively lower postural control scores alongside variable thoracoabdominal configurations, whereas the youngest participant with the lowest gross motor score presented a different distribution of axial, musculoskeletal, and respiratory characteristics. These observations suggest that functional domains did not demonstrate a consistent descriptive pattern within individuals. Rather than reflecting a uniform progression across domains, the dataset illustrates heterogeneous multidimensional profiles within this exploratory sample. For visualization purposes in Figure 2, selected variables were normalized within the cohort to a 0–1 range (min–max scaling), where 0 and 1 represent the lowest and highest observed values within this sample, respectively. This transformation was applied solely for graphical presentation and was not used for statistical inference.

### 3.5. Intra-Rater Measurement Consistency

Intra-rater consistency was evaluated using repeated measurements performed within the same assessment session. Given the small sample size and limited between-subject variability characteristic of this pilot cohort, absolute mean differences between repeated measurements were calculated as a stable indicator of measurement precision. Detailed measurement differences are presented in Table 5.

Angular joint parameters demonstrated minimal variability (mean differences 0.17–1.67°, most ≤1°, maximum 5°), with particularly low absolute differences observed for sacral inclination and ankle dorsiflexion (≤1°). Thoracoabdominal measures exhibited low absolute variability (≤2 cm for circumferences; ≤0.5 cm for thoracic expansion). Subcostal, sternoclavicular, and posterior rib angles showed slightly greater variability in selected cases (maximum difference 5°), consistent with small positional adjustments and physiological factors inherent to pediatric assessment. Respiratory rate differences (maximum 4 breaths/min) reflected expected physiological fluctuation during quiet breathing. In selected parameters (e.g., left hip extension), repeated measurements were identical across trials. Intraclass correlation coefficients were explored; however, due to the very small sample size and minimal variance in several parameters, ICC estimates proved statistically unstable and were therefore not interpreted. Overall, the magnitude and distribution of measurement differences are consistent with acceptable intra-rater consistency within the context of this pilot study.

## 4. Discussion

### 4.1. Principal Findings

This pilot case series provides an integrated description of functional, postural, musculoskeletal, and thoracoabdominal characteristics in children with Joubert syndrome [16]. By combining standardized assessments of gross motor function and postural control with objective measures of joint mobility, pelvic alignment, and thoracic configuration, the study offers a multidimensional descriptive overview of functional organization beyond isolated gross motor performance scores.

Gross motor function varied widely across participants, while postural control scores did not parallel gross motor performance levels within the cohort. Measures of joint range of motion, pelvic alignment, and thoracic configuration also demonstrated substantial inter-individual variability. It should also be acknowledged that the cohort included genetically heterogeneous subtypes of Joubert syndrome, and genotype-related differences may have contributed to the observed variability, although this could not be explored within the constraints of the present sample size. In several participants, relatively preserved gross motor performance coexisted with lower postural control scores, suggesting that task-based motor ability and axial stabilization capacity may reflect distinct descriptive dimensions within this sample.

Taken together, the findings in this exploratory cohort suggest that functional characteristics may not be fully captured by global motor scores alone. These domains may represent distinct dimensions of functional presentation within this cohort. While no inferential conclusions can be drawn, the observed multidimensional variability supports further descriptive characterization of motor function using integrated assessment domains.

In interpreting these findings, it is also important to consider the developmental dimension of axial and respiratory–postural organization. Trunk control and thoracoabdominal coordination undergo progressive maturation across childhood, reflecting ongoing refinement of cerebellar and distributed sensorimotor networks. Given the broad pediatric age range represented in this cohort, part of the observed variability may relate to maturational stage rather than exclusively to syndrome-specific mechanisms.

The variability described in this cohort represents descriptive empirical observations derived from within-sample assessment. The proposed neurodevelopmental interpretation, grounded in developmental motor control models and cerebellar network theory, serves as a conceptual framework for contextualizing these findings rather than as direct mechanistic evidence. Accordingly, the present data should not be interpreted as demonstrating specific causal cerebellar mechanisms but rather as illustrating functional variability that may be compatible with established theoretical models.

### 4.2. Axial Control Beyond Gross Motor Performance

Global gross motor scores such as the GMFM-88 are widely used to quantify motor abilities in children. The GMFM provides a reliable measure of task completion and motor capacity. However, it primarily reflects the quantity of movement rather than the quality of postural organization or movement strategies [17]. Although BARS is not an ataxia-specific neurological scale, it captures functional aspects of impaired coordination and axial instability, which have been described as core features of the motor phenotype in Joubert syndrome [18,19].

Postural control and axial stability represent related but distinct functional constructs. Impairments in balance and trunk control may affect mobility and daily function even in children with similar gross motor scores [20,21,22,23]. Trunk control plays a central role in postural adaptability and movement efficiency. Axial stability is generally understood to involve the integration of cerebellar, brainstem, and supraspinal networks involved in postural control [4,18]. In Joubert syndrome, congenital malformations of cerebellar–brainstem circuitry are known to affect neural networks involved in postural regulation, as described in previous studies.

Within this cohort, postural control scores varied independently of gross motor performance levels, without a consistent descriptive alignment between domains. The descriptive findings indicate that postural stability may represent a functional dimension not fully captured by global motor outcome measures [19,24]. However, previous studies in Joubert syndrome have largely relied on global motor scales and small heterogeneous samples, limiting direct comparison of axial control measures across cohorts.

### 4.3. Postural and Musculoskeletal Characteristics

Postural and musculoskeletal organization is a key component of axial control and functional movement in children with neurodevelopmental disorders [25]. Variations in joint range of motion and pelvic alignment should be interpreted as elements of postural organization rather than as isolated joint-level impairments [21].

In the present cohort, substantial variability in proximal joint mobility was observed, particularly at the hip and shoulder levels. These joints contribute to trunk-extremity interaction during upright postures and transitional movements [26,27]. The observed variability in external rotation may be considered within the broader context of proximal joint organization; however, due to the descriptive and cross-sectional design of this study, it remains unclear whether these patterns reflect compensatory mechanisms, developmental variability, or condition-related characteristics. Therefore, the present findings should be interpreted as descriptive observations rather than evidence of specific underlying mechanisms.

Pelvic alignment, reflected by sacral inclination angle, also varied across participants. Pelvic orientation is known to influence spinal alignment, load distribution, and the mechanical conditions for upright posture [28,29]. Distal joint measures, including ankle dorsiflexion, formed part of the overall postural profiles observed in this cohort. Limitations at the ankle level may influence weight-bearing strategies during standing and supported positions [30,31]. These observations can be discussed in relation to theoretical models of postural control, including the inverted pendulum framework, which describes how distal constraints may shift regulatory demands toward more proximal segments [32,33,34]. However, the present descriptive data do not allow determination of specific compensatory mechanisms or causal relationships. Similar proximal stabilization patterns have been described in other neurodevelopmental conditions [35,36], although their functional significance in Joubert syndrome requires further investigation. It should be noted that musculoskeletal parameters in rare neurodevelopmental disorders are infrequently reported in a structured manner, and existing studies often lack standardized measurement protocols, which limits comparability across reports.

### 4.4. Thoracoabdominal Configuration and Respiration

Thoracoabdominal configuration contributes to axial organization, as the thorax functions as a structural component of posture in addition to its ventilatory role [37,38]. In pediatric and neurological populations, chest wall mobility has been associated with trunk stability and coordination between breathing and movement [39]. Restricted thoracic excursion and altered rib positioning have been described as factors potentially influencing postural mechanics [40].

In this study, thoracic excursion during quiet breathing demonstrated a relatively low and narrow within-cohort range across participants, independent of age or gross motor performance level. Thoracoabdominal measures showed inter-individual variability in rib positioning and thoracic geometry, while respiratory rates remained within a relatively narrow range. The coexistence of limited thoracic excursion and stable respiratory rates should be interpreted cautiously, as the present descriptive design does not allow determination of specific breathing patterns or compensatory mechanisms. Similarly, the coexistence of differing thoracoabdominal configurations and lower postural control scores represents a descriptive observation within this cohort rather than evidence of functional coupling between respiratory mechanics and axial stability.

From a developmental neuroscience perspective, trunk control and respiratory–postural coordination are understood as emerging from cerebellar-brainstem network interactions supporting predictive timing, feedforward regulation, and sensorimotor integration. The cerebellum has been proposed to acquire and update internal models, enabling predictive motor commands and integration of current and anticipated sensory states [41]. Reviews highlight the important contribution of the cerebellum to anticipatory postural adjustments, where timing and prediction are integral to maintaining balance and posture [8]. Cerebellar circuits are also implicated in feed-forward control mechanisms supporting anticipatory motor regulation [42]. Within this framework, the observed multidimensional variability may be compatible with altered network-level integration [38]. Available literature on thoracoabdominal characteristics in Joubert syndrome remains limited, and most prior reports have focused primarily on overt respiratory abnormalities rather than structural or postural parameters, which limits direct comparability with the present descriptive findings. No correlation or inferential analyses were performed, and the present dataset does not permit evaluation of statistical relationships between thoracic configuration and postural control.

The present findings therefore suggest that thoracoabdominal characteristics may represent one descriptive component of the overall functional profile observed in this cohort. However, given the small sample size and cross-sectional design, their specific contribution to axial organization in Joubert syndrome cannot be determined and warrants further investigation.

### 4.5. Clinical Implications

In this exploratory pilot case series, gross motor performance and postural control did not demonstrate a consistent descriptive pattern within this cohort of children with Joubert syndrome. Variability was also observed across postural, musculoskeletal, and thoracoabdominal domains, including among participants with relatively preserved gross motor scores. Thoracic excursion during quiet breathing showed a relatively limited range across participants, irrespective of gross motor performance level.

These descriptive findings highlight that global motor scores may not fully capture all observable functional domains within this cohort. However, given the small sample size and non-controlled design, the present results should not be interpreted as a basis for specific clinical recommendations. Rather, they underscore the potential value of further research exploring multidimensional assessment approaches in larger and controlled studies.

## 5. Study Limitations

This study has several important limitations that should be considered when interpreting the findings. The small sample size (*n* = 6) and feasibility-based recruitment substantially limit statistical precision and preclude inferential analyses. The cross-sectional, non-controlled design limits internal validity and prevents causal interpretation. The absence of a control group does not allow differentiation between features potentially specific to Joubert syndrome and patterns that may also occur in other neurodevelopmental conditions. Recruitment through a national family meeting and voluntary participation may have introduced selection bias, as families engaged in organized support activities may not fully represent the broader clinical population. This limits external validity and generalizability. All assessments were conducted by a single non-blinded examiner. Although intra-rater consistency was explored descriptively, the use of a single examiner introduces the possibility of systematic measurement bias, particularly in relation to thoracoabdominal angular parameters, which were applied as exploratory geometric descriptors and are not widely standardized within pediatric biomechanical assessment literature. The absence of independent verification or blinded replication further limits external validation of these measures. Additionally, postural and thoracoabdominal parameters were analyzed without comparison to age-matched normative data. Given the exploratory nature and limited pediatric standardization of surface-based thoracoabdominal angular measures, their interpretation is restricted to descriptive within-cohort characterization. The wide age range of participants (2–11 years) introduces substantial developmental heterogeneity. Axial control, postural organization, thoracic mobility, and respiratory–postural integration undergoes progressive maturation across childhood. Maturational stage may therefore represent a potential confounder, as age-related neurodevelopmental refinement of cerebellar and sensorimotor networks could independently influence the observed functional patterns. This limits comparability between participants and restricts interpretation to descriptive within-cohort characterization. The cohort also included genetically heterogeneous subtypes of Joubert syndrome. Given the very small sample size, genotype-phenotype associations could not be explored, and genetic variability may have contributed to the observed functional heterogeneity within the cohort. Taken together, these limitations indicate that the present findings should be regarded as exploratory and descriptive rather than confirmatory.

## 6. Future Directions

Future research should aim to replicate these findings in larger, multicenter cohorts to improve statistical robustness and external validity. Inclusion of appropriate comparison groups, such as children with other cerebellar disorders or typically developing controls, would allow clarification of syndrome-specific versus nonspecific functional patterns. Longitudinal study designs will be particularly important to examine developmental trajectories of axial control, postural organization, and thoracoabdominal characteristics across childhood. Repeated multidimensional within-subject assessment may help differentiate maturational processes from condition-related functional patterns and refine phenotypic characterization over time. Integration of structural and functional neuroimaging metrics could further explore potential associations between cerebellar morphology, network connectivity, and multidimensional functional profiles. In addition, phenotype-informed stratification may support more individualized rehabilitation planning, particularly in identifying children whose axial stabilization or thoracoabdominal coordination warrants targeted intervention. Standardized measurement protocols and integration of objective biomechanical or respiratory analyses may further refine understanding of multidimensional functional organization in Joubert syndrome. Prospective, adequately powered studies will be required to formally examine the hypotheses emerging from this exploratory analysis.

## 7. Conclusions

This exploratory, cross-sectional case series provides a multidimensional descriptive characterization of functional features in a small cohort of children with Joubert syndrome. Variability across motor, postural, musculoskeletal, and thoracoabdominal domains was not fully reflected by global gross motor scores alone. Given the small sample size and non-controlled design, these findings should be interpreted cautiously. The proposed multidimensional framework should be regarded as an exploratory conceptual approach rather than a redefinition of clinical assessment and requires longitudinal and multicenter validation to clarify its mechanistic and clinical relevance.

## Figures and Tables

**Table 1:** Demographic, Clinical, and Genetic Characteristics of the Study Cohort.

Variable	P1	P2	P3	P4	P5	P6
Age (years)	9	10	11	5	2	6
Sex	M	F	M	M	M	F
Height (cm)	127	136	139	120	98	117
Weight (kg)	38	33	32	25	14	22
MRI molar tooth sign	Yes	Yes	Yes	Yes	Yes	Yes
Gene (if available)	PIBF1	NPHP1	TMEM67	TMEM67	TMEM67/CC2D2A	NAGA

Abbreviations: M, male; F, female; MRI, magnetic resonance imaging; cm, centimeters; kg, kilograms.

**Table 2:** Gross Motor Function and Postural Control.

Variable	P1	P2	P3	P4	P5	P6
GMFM-88 total score (%)	24.6	84.5	74.6	82.2	10.2	79.9
BARS total score	19	4	6	11	28	5

Abbreviations: GMFM-88, Gross Motor Function Measure-88; BARS, Balance Assessment Rating Scale.

**Table 3:** Postural and Musculoskeletal Characteristics.

Variable	P1	P2	P3	P4	P5	P6
Shoulder external rotation—right (°)	20	100	110	120	110	110
Shoulder external rotation—left (°)	20	100	110	120	110	110
Hip extension (°)	0	20	20	0	20	20
Hip external rotation—right (°)	90	90	60	90	45	50
Hip external rotation—left (°)	90	80	70	90	80	55
Ankle dorsiflexion—right (°)	0	5	10	28	0	0
Ankle dorsiflexion—left (°)	0	0	10	26	0	0
Sacral inclination angle (°)	20	18	18	25	15	15

Abbreviations: °, degrees.

**Table 4:** Thoracoabdominal Configuration and Respiratory Mobility.

Variable	P1	P2	P3	P4	P5	P6
Chest circumference (cm)	83	70	68	65	54	60
Abdominal circumference (cm)	74	64	66	66	51	60
Thoracic expansion during quiet breathing (cm)	1.5	2	2	1.5	0.5	2
Respiratory rate during quiet breathing (breaths/min)	16	12	12	18	12	15
Subcostal angle (°)	85	135	110	120	140	145
Sternoclavicular angle—right (°)	128	110	122	125	145	115
Sternoclavicular angle—left (°)	128	110	135	130	130	125
Posterior rib angle—right (°)	70	55	65	70	75	60
Posterior rib angle—left (°)	60	70	60	60	75	75

Abbreviations: cm, centimeters; °, degrees; breaths/min, breaths per minute.

**Table 5:** Intra-Rater Consistency of Repeated Measurements.

Parameter	Mean Difference (M1 − M2)	Range
Shoulder external rotation—right (°)	0.67	0–2
Shoulder external rotation—left (°)	1.67	0–5
Hip extension—right (°)	0.33	0–2
Hip extension—left (°)	0.00	0–0
Hip external rotation—right (°)	1.17	0–5
Hip external rotation—left (°)	0.83	0–5
Ankle dorsiflexion—right (°)	0.17	0–1
Ankle dorsiflexion—left (°)	0.33	0–2
Sacral inclination angle (°)	0.33	0–1
Chest circumference (cm)	0.33	0–1
Abdominal circumference (cm)	0.83	0–2
Thoracic expansion during quiet breathing (cm)	0.83	0–0.5
Respiratory rate (breaths/min)	1.50	0–4
Subcostal angle (°)	1.83	0–5
Sternoclavicular angle—right (°)	0.50	0–2
Sternoclavicular angle—left (°)	2.50	0–5
Posterior rib angle—right (°)	2.00	0–5
Posterior rib angle—left (°)	1.67	0–5

Abbreviations: M1, first measurement; M2, second measurement; °, degrees; cm, centimeters; breaths/min, breaths per minute.

## Data Availability

The data presented in this study are not publicly available due to ethical and privacy restrictions. Anonymized data may be made available from the corresponding author upon reasonable request.

## Abbreviations

JS	Joubert syndrome
MRI	Magnetic resonance imaging
GMFM-88	Gross Motor Function Measure-88
PEDI	Pediatric Evaluation of Disability Inventory
BARS	Balance Assessment Rating Scale
ICF	International Classification of Functioning
ICC	Intraclass correlation coefficient
